# Effect of COVID-19 on epidemiological characteristics of road traffic injuries in Suzhou: a retrospective study

**DOI:** 10.1186/s12873-021-00483-7

**Published:** 2021-07-26

**Authors:** Wenjuan Huang, Qi Lin, Feng Xu, Du Chen

**Affiliations:** 1 Department of Critical Care Medicine, the First Affiliated Hospital of Soochow University, Suzhou City, Jiangsu Province China; 2 Suzhou Emergency Center, Suzhou City, Jiangsu Province China; 3 Department of Emergency Medicine, the First Affiliated Hospital of Soochow University, Suzhou City, Jiangsu Province China

**Keywords:** COVID-19, Road traffic injuries, Suzhou, Epidemiology

## Abstract

**Background:**

To present the new trends in epidemiology of road traffic injuries (RTIs) during the Coronavirus disease 2019 (COVID-19) pandemic in Suzhou.

**Methods:**

Pre-hospital records of RTIs from January to May in 2020 and the same period in 2019 were obtained from the database of Suzhou pre-hospital emergency center, Jiangsu, China. Data were extracted for analysis, including demographic characteristics, pre-hospital vital signs, transport, shock index, consciousness, pre-hospital death. A retrospective study comparing epidemiological characteristics of RTIs in Suzhou during the 5-month period in 2020 to the parallel period in 2019 was performed.

**Results:**

A total of 7288 RTIs in 2020 and 8869 in 2019 met inclusion criteria. The overall volume of RTIs has statistical difference between the 2 years (*p* < 0.001), with fewer RTIs in 2020 compared with 2019*.* Electric bicycle related RTIs increased during the pandemic (2641, 36.24% vs 2380, 26.84%, *p* < 0.001), with a higher incidence of RTIs with disorder of consciousness (DOC) (7.22% vs 6.13%, *p* = 0.006).

**Conclusions:**

Under the impact of COVID-19, the total number of RTIs in Suzhou from January to May 2020 decreased. This observation was coupled with a rise in electric bicycle related injuries and an increase in the incidence of RTIs with DOC.

**Supplementary Information:**

The online version contains supplementary material available at 10.1186/s12873-021-00483-7.

## Background

The Global status report on road safety 2018, launched by WHO in December 2018, highlights that the number of annual deaths resulting from RTIs has reached 1.35 million. The RTIs are the eighth leading cause of death for people of all ages and the leading killer of children and young adults aged 5–29 years [1]. Low- and middle-income countries bear the greatest burden of road traffic fatalities and injuries [1].

In China, the RTIs are the second cause of all types of injuries that lead to emergency department visits, constituting 21% of all injuries, and have become the leading cause of injury deaths [2]. The highest mortality of RTIs occurred among young adults aged 20–45 years, particularly males, and in rural areas [2].

The COVID-19 is caused by the severe acute respiratory syndrome coronavirus 2 (SARS-CoV-2) [3]. This virus is believed to be transmitted by aerosols and/or droplets, and has showed strong infectivity and pathogenicity [3, 4]. The institution of interventions including traffic restriction, social distancing, lockdowns were the effective strategy against acquiring and spreading COVID-19 [5].

The COVID-19 pandemic has significantly affected all walks of life, including the volume and nature of RTIs. There was a significant decrease in the number of road traffic collisions during the COVID-19-related period of societal restrictions and lockdown [6]. Whilst a research has showed trauma admissions decreased in the lockdown phase with an increased incidence of road traffic accidents [7]. A reduction in the number of road traffic accident fatalities was observed during the lockdown period [8–11]. Furthermore, the mandated societal lockdown policies led to reduction in road traffic accidents resulting in non-serious or no injuries but not those resulting in serious or fatal injuries [12]. In addition, Kunal Rajput et al. have showed that the road traffic collisions involving a car significantly reduced during lockdown, conversely, bike-related road traffic collisions significantly increased [13].

Few studies have showed the characteristics of RTIs in Suzhou during the COVID-19 outbreak. The aim of this study was to present the new trends in epidemiology of RTIs in Suzhou during the COVID-19 pandemic.

## Methods

### Study design

In China, the emergency medical service (EMS) system consists of pre-hospital emergency system and hospital emergency department (ED). Pre-hospital emergency treatment refers to the emergency treatment for critically ill patients outside the hospital and plays an irreplaceable role in meeting people’s daily emergency needs, providing medical security for major activities and dealing with emergencies and natural disasters [14]. The hospital ED is the core of the EMS system, and it is a professional department dealing with acute and critical treatment, public emergencies, mass trauma events, natural disasters, and collective poisoning events [14].

**Fig. 1:**
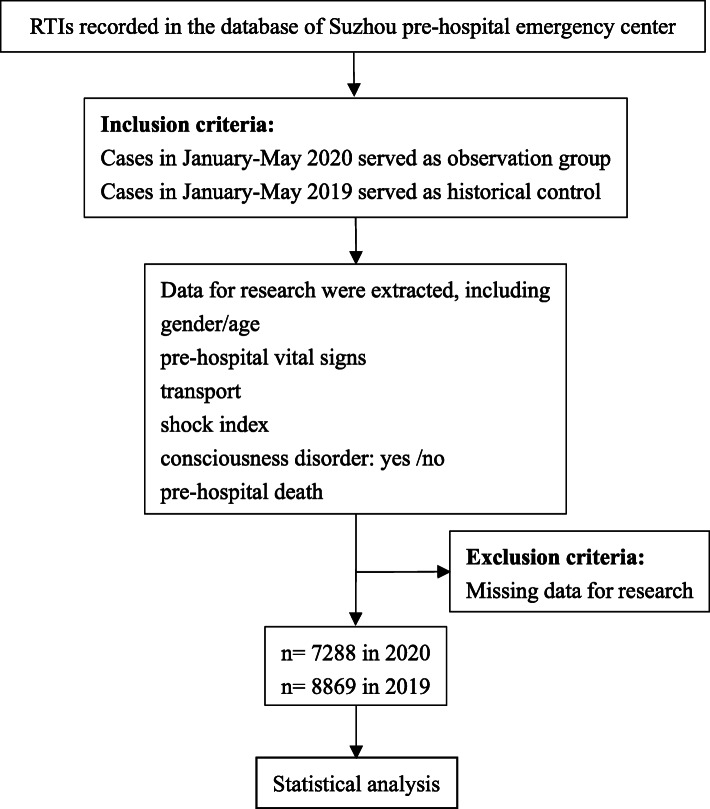
Methodology of our study-depicting research

In this study, the pre-hospital records were obtained from the database of Suzhou pre-hospital emergency center, Jiangsu province, China. Cases were selected after taking into account the inclusion and exclusion criteria (Fig. 1). The RTIs from January to May in 2020 were included for analysis. The RTIs from the same period in 2019 were used as historical control. The variables, including demographic characteristics, pre-hospital vital signs, transport, injury pattern (shock, DOC, pre- hospital death) were collected. Shock was defined as a shock index (pulse rate /systolic blood pressure)>1, and DOC is defined as Glasgow Coma Scale (GCS) < 15 points. A retrospective analysis was performed.

The primary outcome of interest was the trend in the number of RTIs. Secondary outcomes included changes in transport, injury nature, and pre-hospital mortality.

### Statistical analysis

Categorical data were expressed as frequency (percentage), and compared using chi-square test. Continuous variables were tested for normality using Shapiro–Wilk test. All of the continuous variables failed to conform to normality were expressed as median (IQR) and compared using Mann-Whitney test. Statistical analyses were completed with STATA 15.0, the graph was plotted in MS Excel. Statistical significance was defined as a 2-sided *P* value of < 0.05.

## Results

**Fig. 2:**
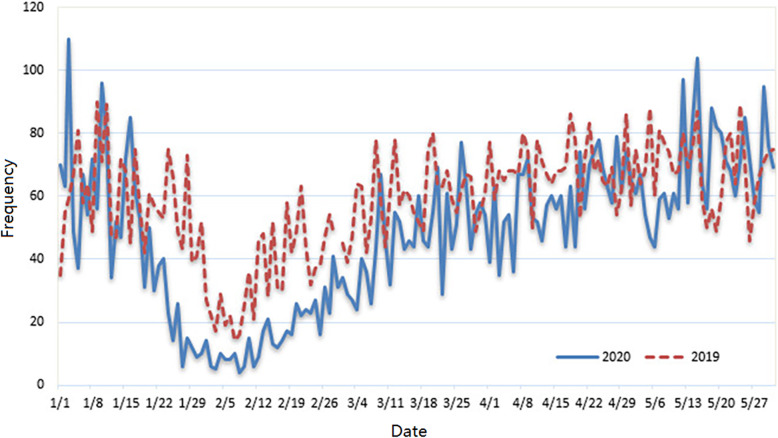
The daily number of RTIs in Suzhou in January–May 2020 and January–May 2019

A total of 7288 RTIs in 2020 and 8869 in 2019 met inclusion criteria (Table 1). The overall volume of RTIs has statistical difference between the 2 years (*p* < 0.001), with fewer RTIs in 2020 compared with 2019*.* In February of these 2 years, the number of RTIs was lowest. The daily number of RTIs in Suzhou in January–May 2020 and January–May 2019 were respectively showed in Fig. 2. Comparatively, the trend in the number of RTIs reports an overall reduction in 2020 when compared to 2019. The most marked reduction occurred in late January and February 2020 when compared to year 2019.

**Table 1:** Characteristics of RTIs in Suzhou in January–May 2020 and January–May 2019

	2019 (***n*** = 8869)	2020 (***n*** = 7288)	p value
**Number of RTIs, n (%)**			< 0.001
Jan.	1834(20.68)	1462(20.06)	
Feb.	996(11.23)	475(6.52)	
Mar.	1831(20.65)	1452(19.92)	
Apr.	2073(23.37)	1777(24.38)	
May	2135(24.07)	2122(29.12)	
**Electric bicycle related RTIs, n (%**)	2380(26.84)	2641(36.24)	< 0.001
**Gender**			0.050
Female, n (%)	4088(46.09)	3247(44.55)	
Male, n (%)	4781(53.91)	4041(55.45)	
**Age, years (SD)**	47(27)	48(27)	0.045
**Vital signs**			
PR, bpm (SD)	83(14)	82(15)	0.025
RR, bpm (SD)	18(3)	18(2)	0.385
SBP, mmHg (SD)	133(28)	134(28)	0.436
DBP, mmHg (SD)	82(17)	81(17)	0.248
**Injuries**			
Shock, n (%)	245(2.76)	214(2.94)	0.508
DOC, n (%)	544(6.13)	526(7.22)	0.006
Death, n (%)	91(1.03)	79(1.08)	0.720

Abbreviations: *RTIs* road traffic injuries, *PR* pulse rate, *RR* respiratory rate, *bpm* beats per minute, *SBP* systolic blood pressure, *DBP* diastolic blood pressure, *SD* standard deviation, *DOC* disorder of consciousness

Electric bicycle related RTIs increased during the pandemic (2641, 36.24% vs 2380, 26.84%, *p* < 0.001), with a higher incidence of RTIs with DOC (7.22% vs 6.13%, *p* = 0.006).

Compared with the RTIs in 2019, the median age of cases in 2020 was 1 year older (*p* = 0.045). Regarding gender, shock and death, no significant differences were detected between the two time periods (*p* = 0.050, *p* = 0.508 and *p* = 0.720 respectively).

## Discussion

The outbreak of COVID-19 has caused global concerns. Currently, the therapeutics for COVID-19 including supporting treatment, drugs, vaccines, while control and prevention are other strategies to reduce the transmission within China and elsewhere [15, 16]. The local government in Wuhan announced the suspension of public transportation, including the closure of railway stations, highways and airports on January 23, 2020, to prevent further disease spread [17]. Consequently, Hubei province was placed under lockdown approximately 3 weeks after the start of COVID-19 outbreak [18]. The Chinese government made great efforts to control the flow of people. Shopping malls and other entertainment activities were closed, in-person classes were replaced by online ones, public transport was restricted, public gatherings were banned and routine health checks were carried out in order to prevent the spread of SAR-CoV2 right after Wuhan shutdown [19].

Due to the impact of COVID-19 and relevant control measures, the way people travel has also undergone subtle changes. Indeed, some new trends have emerged in the epidemiology of RTIs during pandemic in Suzhou.

In the present study, the total volume of RTIs decreased during the COVID-19 pandemic, which is consistent with previous studies [6, 12, 13]. The decline is mostly attributed to the above restrictions. People might be more inclined to perform activities at home which would result in less car traffic and less congestion during peak hours [20]. This could account for reduced traffic burden, resulting in a decrease in the number of RTIs.

Furthermore, in this research, the most marked reduction occurred in late January and February 2020 when compared to year 2019. This is due to the fact that domestic pandemic was most severe in late January and February 2020 and under the strictest control. In February 2019, the volume of RTIs was also the lowest throughout the year. This is attributed to Spring Festival holiday in early February. Large numbers of migrant workers have left Suzhou and returned to hometowns, which resulting in declines in traffic burden and RTIs.

Whilst in this study, electric bicycle related RTIs increased during the pandemic, with a higher incidence of RTIs with DOC. China is a large consumer of electric bicycle. In order to avoid close contact during the pandemic, people preferred electric bicycle to public transport, which directly led to an increase in electric bicycle related RTIs. This result is consistent with that of Kunal Rajput et al. [13]. They have reported an increase in bike- or pushbike related road traffic collisions during lockdown, which is likely due to the fact that riding a bike became the chosen mode of exercise for many people in warmer weather while keeping to the statutory 2 m distance. In addition, the traffic volume has decreased but the cycling speed may have increased due to lower congestion on roads.

Meanwhile, in the event of road traffic accidents, riders without wearing helmets are vulnerable to head injury [21, 22]. Generally, in road traffic collisions, the DOC is often associated with head injury. During the study period, electric bicycle riders in Suzhou generally did not wear helmets, resulting in a rise in the incidence of RTIs with DOC. Therefore, Suzhou government has carried out the “helmet-belt” safety protection activity, to increase the use of helmets among electric bicycle riders and seatbelts among car occupants, which is very necessary for electric bicycle riders to reduce head injury.

In the present study, the mortality of RTIs reported in January–May 2020 was similar to that reported during the parallel period in 2019. This result is inconsistent with those reported in previous studies [9–11]. In the above studies, fatalities resulting from road traffic accidents exhibit a significant decrease, possibly because of the restrictions (such as lockdowns) that were imposed to contain the spread of the COVID-19 pandemic in 2020 [9–11]. In addition, the deaths referred in our study were those occurring in pre-hospital, deaths occurring in hospitals were not included, which might lead to underestimated fatalities resulting from RTIs.

Compared with the RTIs in 2019, the median age of cases in 2020 was 1 year older (*p* = 0.045). This finding deserves further study when more data will become available. Concerning gender and shock, our analysis did not reveal any statistically significant differences.

The present study has some limitations. First, only the data of the 5 months from January to May were analyzed, but the medium - and long-term impacts of COVID-19 epidemic on RTIs were not clear. Second, Suzhou is a prefecture-level city in China, the involvement of just one center resulted in a smaller sample size. This study could be repeated with a larger sample size, which would add weight to our conclusions. Third, there are many factors affecting RTIs in reality, the influence of other confounding factors cannot be completely excluded from the historical data only. In the cases of minor injuries, emergency services may not be activated and therefore no pre-hospital records are available. The present study potentially underestimates the true number of RTIs.

## Conclusions

Under the impact of COVID-19, the total number of RTIs in Suzhou from January to May 2020 decreased. This observation was coupled with a rise in electric bicycle related injuries and an increase in the incidence of RTIs with DOC.

## Supplementary Information

**Additional file 1.**

## Data Availability

All data generated or analyzed during this study are included in this article and its supplementary information files.

## Abbreviations

RTIs: road traffic injuries
COVID-19: Coronavirus disease 2019
SARS-CoV-2: severe acute respiratory syndrome coronavirus 2
EMS: emergency medical service
ED: emergency department
GCS: Glasgow Coma Scale
PR: pulse rate
RR: respiratory rate
bpm: beats per minute
SBP: systolic blood pressure
DBP: diastolic blood pressure
SD: standard deviation
DOC: disorder of consciousness
